# Three dimensional characterization of GaN-based light emitting diode grown on patterned sapphire substrate by confocal Raman and photoluminescence spectromicroscopy

**DOI:** 10.1038/srep45519

**Published:** 2017-03-30

**Authors:** Heng Li, Hui-Yu Cheng, Wei-Liang Chen, Yi-Hsin Huang, Chi-Kang Li, Chiao-Yun Chang, Yuh-Renn Wu, Tien-Chang Lu, Yu-Ming Chang

**Affiliations:** 1 Dept. of Photonics & Institute of Electro-Optical Engineering, National Chiao Tung University, Hsinchu 30050, Taiwan; 2 Center for Condensed Matter Sciences, National Taiwan University, 10617, Taipei, Taiwan; 3 Graduate Institute of Photonics and Optoelectronics and Department of Electrical Engineering, National Taiwan University, Taipei, 10617, Taiwan

## Abstract

We performed depth-resolved PL and Raman spectral mappings of a GaN-based LED structure grown on a patterned sapphire substrate (PSS). Our results showed that the Raman mapping in the PSS-GaN heterointerface and the PL mapping in the In_x_Ga_1−x_N/GaN MQWs active layer are spatially correlated. Based on the 3D construction of E_2_(high) Raman peak intensity and frequency shift, V-shaped pits in the MQWs can be traced down to the dislocations originated in the cone tip area of PSS. Detail analysis of the PL peak distribution further revealed that the indium composition in the MQWs is related to the residual strain propagating from the PSS-GaN heterointerface toward the LED surface. Numerical simulation based on the indium composition distribution also led to a radiative recombination rate distribution that shows agreement with the experimental PL intensity distribution in the In_x_Ga_1−x_N/GaN MQWs active layer.

In this century InGaN/GaN light emitting diodes (LEDs) have become the most widely used optoelectronic devices for solid-state lighting. Its advantages include a widely tunable range of emitting wavelengths from ultraviolet (UV) to near infrared (NIR) and a high electric-optical conversion efficiency. The material research in nitride semiconductors has also led to successful developments in optoelectronic device applications, such as laser diodes, photodetectors, solar cells, and power electronics^1^,^2^,^3^,^4^,^5^,^6^. Specifically, InGaN compound semiconductors exhibit large oscillator strength, large exciton binding energy, high thermal conductivity, good mechanical stability, and intriguing optoelectronic properties, making it suitable for high power lighting applications^7^. However, due to the phenomenon of efficiency droop, the efficiency of GaN-based LEDs is suppressed with increasing injection current. The large defect and dislocation density induced by the large lattice mismatch between GaN and hetero-substrates has also limited GaN-based light emitting devices for high current injection operations. It has become increasingly important to overcome the droop problem as LED devices are requested to maintain the same or even higher output power in decreased chip sizes^8^,^9^.

Another important factor limiting the development of GaN-based devices is the lack of native substrates for GaN epitaxy. Commercial GaN-based LEDs are typically grown on c-plane sapphire substrate by hetero-epitaxy approach. Due to the mismatch between the sapphire and GaN lattices, this approach results in a large defect and dislocation density in the subsequently grown GaN epilayer^10^,^11^. Recently, one alternative and clever approach to suppress the defect and dislocation densities is the use of patterned sapphire substrate (PSS). This novel approach not only improves the crystal quality but also enhances the light extraction efficiency of GaN-based LED devices^12^,^13^. On the other hand, the lattice mismatch can also induce built-in strain and internal electric field, so called the quantum-confined Stark effect (QCSE), in the c-plane direction^14^,^15^. Note that QCSE can cause the band bending and the separation of the electron and hole wave functions in the quantum-well (QW) region, and results in the suppression of radiative recombination efficiency of GaN-based LED devices.

In order to further improve the GaN-based LED efficiency, many groups have designed and analyzed GaN-based LEDs with various PSS structures, which may differ in shape, height, or period of the designed pattern^16^,^17^,^18^. Spatially resolved micro-Raman spectroscopy has also been used to observe the in-plane strain distribution by measuring the E_2_(high) Raman peak variation in the GaN epilayer grown on the top of the flat or cone areas of PSS^19^. A properly designed PSS can mitigate the compressive strain in GaN epilayer and suppress the dislocation density by bending the threading dislocation (TD) along the tilted surface of PSS^17^. The strain variation not only causes the spatial difference of QCSE, but also contributes to indium fluctuation in the InGaN/GaN multiple quantum wells (MQWs). The efficiency droop behavior in GaN-based LEDs differs with the level of effective band gap fluctuation. This band gap fluctuation not only enhances the localization effect, which increases the carrier wave function overlap and prevent carrier leakage through dislocations, but also improves the internal quantum efficiency (IQE)^20^. Therefore the choice of substrate is expected to play a significant role in determining many of the LEDs’ characteristics including dislocation, strain, and indium distribution in the MQWs. The PL emission pattern for GaN-based LEDs has been reported to be quite non-uniform by several groups^20^,^21^,^22^. It was generally believed that the non-uniformity influences the LED efficiency. A rapid and nondestructive three dimensional (3D) characterization of GaN epilayer grown on hetero-substrate such as c-plane sapphire would help to identify the possible affecting parameters.

In this study, the GaN-based LED structure was grown without the p-GaN layer in order to observe the optical properties of MQWs and V-shape pits directly. By performing spatial-resolved photoluminescence (PL) and Raman spectral mapping, we propose to directly investigate and reveal the possible correlation among PSS, strain distribution in GaN epilayer, and PL emission in InGaN/GaN MQWs. We believe that our research method used in this study may offer a great opportunity in evaluating the performance of new PSS design and provide new insight to realize the efficiency droop effect of GaN-based LEDs in the near future.

## Experiment

**Figure 1:**
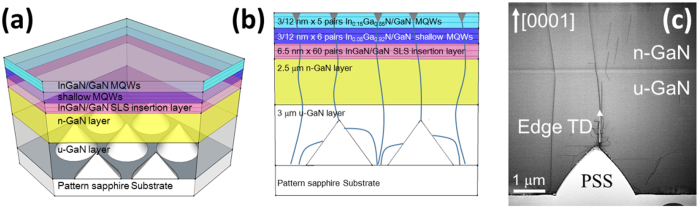
(**a**) Three-dimension schematic and (**b**) two-dimensional cross-section schematic of the epitaxial GaN layers grown on PSS, showing the thickness of each layer. The solid blue lines indicate the propagation of threading dislocations, which end up with V-shaped pits on the surface. (**c**) TEM cross-section image of epitaxial GaN layers around a PSS cone.

The LED structure used in this work was grown on a c-plane PSS by metal organic chemical vapor deposition (MOCVD). As shown in Fig. 1, the epitaxial structure consists of a 20 nm-thick GaN nucleation layer, a 3 μm-thick un-doped GaN (u-GaN) layer, and a 2.5 μm-thick Si-doped n-GaN layer on top of the PSS. On top of the n-GaN layer are 60 pairs of superlattice layers (SLs), which assist the V-pits formation along the TDs. The final layer consists of an InGaN/GaN multi-quantum wells (MQWs) active layer^23^. There are two groups of quantum wells, each with a different indium composition. The first group consists of six wells and has 8% indium composition, while the second group consists of the following nine wells with 15% indium composition. In both groups, the thickness of InGaN wells is 2.5~3.0 nm, and the thickness of GaN barriers is 12 nm. The period of SLs pair is approximately 6.5 nm, and the indium composition is about 5%. The thickness of GaN between the SLs and MQW is about 65 nm. The p-GaN layer was not grown in order to investigate the spatial properties of MQWs.

The spatial emission properties and strain distribution in the MQWs were observed by depth resolved PL and Raman mapping measurements. The PL and Raman mappings were performed on a home-built laser scanning confocal microscope, using 375 nm and 532 nm laser excitation, respectively. An Olympus MPlan, 100×, NA0.9 was used to focus the laser light onto the sample and achieved a lateral resolution of ~200 nm for PL and ~300 nm for Raman near the sample surface. The axial resolution near the surface of the sample was ~700 nm for PL and ~1 μm for Raman. Both axial and lateral resolution degrade with increasing imaging depth, as a result of refraction index mismatch between the objective immersion and the sample^24^,^25^,^26^. For PL mapping, the on-sample power was 20 μW, and for Raman mapping was ~100 mW. The PL and Raman signals collected by the same objective was focused onto the aperture of an optical fiber. For PL measurements, we used a home-built spectrometer which is fitted with a thermoelectric cooled CCD, and has a spectral resolution of ~1 nm. For Raman mapping, we used a high resolution spectrometer system (Jobin-Yvan FHR640 + LN_2_ cooled Symphony CCD), with a spectral resolution of ~0.3 cm^−1^. For laser scanning confocal imaging used in this study, the confocal pinhole blocks out light not coming directly from the excitation focal volume, so the measured PL or Raman intensity is minimally effected by the light extraction of LED structures, and is representative of the direct emission from the focal volume. Transmission electron microscopy (TEM) images have shown that the TDs can originate from the tip of the cone and the flat region of PSS (Fig. 1(c)). These TDs propagate through the GaN epilayer and terminate with V-shaped pits at the surface of MQWs. However, TEM images are normally taken in a restricted field of sight and the measurement is destructive. The depth resolved confocal Raman and PL mapping used in this study can nondestructively produce a 3D characterization of LED structures, and identify the relationship between PSS, V-shaped pits, strain and indium composition in the InGaN/GaN MQWs.

## Results and Discussion

**Figure 2:**
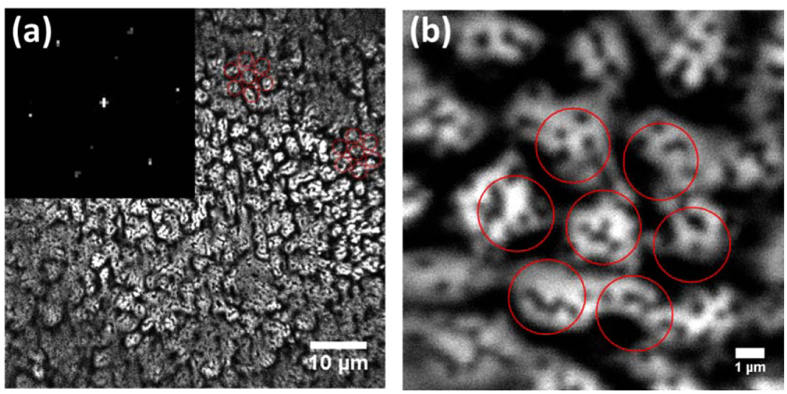
Spatial PL intensity mapping images of the InGaN/GaN MQWs layer in (**a**) a large scale of about 60 × 60 μm^2^ and in (**b**) a scale of one full cell of hexagonal PSS. The inset in (**a**) shows the 2D Fourier transform of the PL image. The red circles indicate the hexagonal cone pattern of PSS.

Figure 2(a) shows the spatial PL intensity mapping of our sample at the MQWs layer. The emission patterns seemed to be randomly organized at the first glimpse. However, after the PL intensity mapping was Fourier transformed into the image shown in the inset of Fig. 2(a), a hexagonal pattern that follows the PSS pattern is clearly observed, indicating the PL emission intensity is strongly correlated to the underneath substrate pattern. This correlation can be further illustrated by adding the patterns of PSS (red circles) to the PL emission intensity mapping, particularly in the enlarged view of PL mapping (Fig. 2(b)). The red circles in both Fig. 2(a,b) represent the position of the cones of PSS. It is interesting to note that most of the bright regions are located above the cone area of PSS, and several small dark points appear in these bright regions. In contrast, the flat regions between PSS cones show weaker PL emission intensity. The reason for those weak and dark PL emission intensity areas can be attributed to the lower carrier concentration due to the delocalized states or the presence of nonradiative recombination centers.

**Figure 3:**
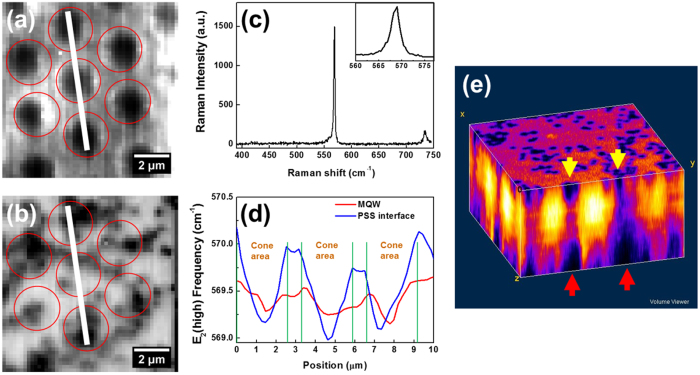
The mapping of GaN E_2_(high) phonon peak intensity at (**a**) PSS-GaN interface and (**b**) InGaN/GaN MQWs layer. (**c**) A typical Raman spectrum collected for the mapping results of (**a**,**b**). Inset shows a zoom-in view of the E_2_(high) peak. (**d**) Plot of E_2_(high) phonon peak position variation along the white lines indicated in (**a**,**b**). Note that PSS cone areas are marked for reference. (**e**) 3D mapping of E_2_(high) phonon peak intensity in a volume of 11 μm (L) × 11 μm (W) × 6 μm (H), where the red and yellow arrows mark the positions of the PSS cones and the V-shaped pits, respectively.

The relation between PSS and emission uniformity was further investigated using confocal Raman spectroscopy, which allows the strain distribution in a specific layer of the sample to be resolved. We performed Raman spectral mapping at the depth of the substrate interface and the MQW layer. Figure 3(a,b) show the GaN E_2_(high) intensity at the substrate interface and the MQW layer. In Fig. 3(b), the PSS patterns appear as the patterns of the dark regions because there are no GaN inside the cones of the PSS. Nevertheless, due to the degradation of the laser focus deep inside the sample, GaN spectra are still obtained inside the cone area, though at a much lower intensity^25^,^26^. Figure 3(c) shows a sample spectrum of the GaN E_2_(high) peak, with an inset showing a zoomed-in view of the E_2_(high) peak. To investigate the strain distribution, we determine the GaN E_2_(high) peak position to a precision of ~0.1 cm^−1^ by curve-fitting each point in the spectral mapping. In Fig. 3(d) we plot the variation of the GaN E_2_(high) peak position along the white lines in Fig. 3(a,b). Since the phonon frequency shift of E_2_(high) Raman peak is sensitive to the biaxial strain along the c-plane of GaN^27^,^28^, Fig. 3(d) effectively shows the strain distribution at the InGaN/GaN MQWs layer and the PSS-GaN interface layer. For unstrained bulk GaN, the E_2_(high) phonon frequency is 568 cm^−1^ 29. In Fig. 3(d), the phonon frequency shift of E_2_(high) Raman peak ranges from 569 cm^−1^ to 570 cm^−1^. As a result, our sample exhibits significant compressive strain from the PSS interface to the MQWs layer. Figure 3(d) shows that the average compressive strain at the PSS interface is larger than that of the MQWs, indicating the gradual strain relaxation during the growth of GaN on PSS^26^. In addition, the fluctuation pattern of compressive strain at the PSS interface exactly corresponds to the pattern of PSS, and the lower E_2_(high) phonon frequency indicating a smaller compressive strain was measured at the cone area. Although the residual strain fluctuation in the MQWs is not as large as that in the substrate interface, the correlation between the strain fluctuation in the MQWs and PSS is still observed. The residual strain in the MQWs above the cone area is relatively smaller than the compressive strain above the flat area of PSS. The smaller strain condition of MQWs above the cone areas corresponds to the higher PL emission intensity areas shown in Fig. 2(b). Figure 3(e) shows a 3D construction of E_2_(high) phonon peak intensity, with the axial dimension adjusted for the depth compression effect caused by the refraction index mismatch between the objective immersion medium (air) and GaN^24^,^25^. In our sample, the compression factor of 2.35 was experimentally determined by comparing the depth resolved image with a direct sectional image of the sample. The dark regions near the substrates (red arrows) reveal the cone shapes of PSS, and the dark spots at the surface (yellow arrows) represent the V-shaped defects that exhibit weaker Raman signals. Therefore, it can be clearly seen that the formation of V-shaped pits and dislocation distribution can be traced back to the cone tips of PSS. The accumulation of dislocation can form the V-pits and lead to the relaxation of compressive strain above the cone area of PSS^30^.

**Figure 4:**
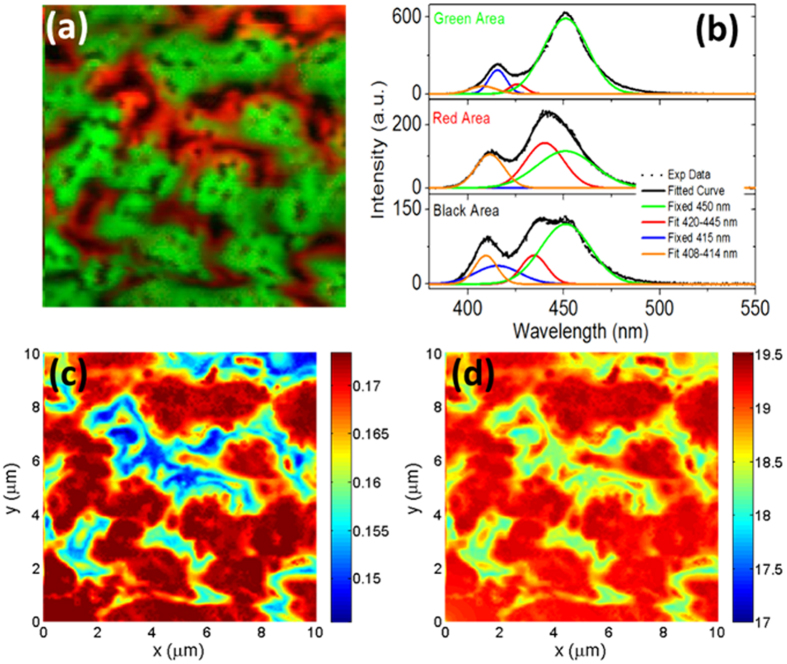
(**a**) The color mapping of the major and minor PL peaks derived from the curve fitting result of the PL spectral mapping. The green represents the intensity of the major peak fixed at 450 nm, and the red represents the intensity of the minor peak with wavelength varying between 420 and 445 nm. (**b**) Selected PL spectra and their curve-fitting results to represent the green, red, and black areas shown in (**a**). Note that the green and blue curves indicate the major PL peak contributions, while the red and orange curves are for the minor PL peak contributions. In the black area PL spectra intensities are low, and both minor PL peaks are more blue-shifted. (**c**) Indium composition distribution in the InGaN/GaN MQWs is estimated based on the obtained major and minor PL peak wavelengths and intensities. The values on the calibration bar label the indium composition in fraction. (**d**) Mapping of the simulated radiative recombination rate in the MQWs. The values on the calibration bar denote the logarithm of the recombination rate in the unit of cm^−3^ s^−1^.

The results of the complete PL spectrum mapping and the 2D characteristics simulation in the area of 10 × 10 μm^2^ are shown in Fig. 4. Here, the PL emission spectra are curve-fitted by four Gaussian PL peaks, where the peak positions of two major peaks are fixed at 415 and 450 nm while the other two corresponding minor peaks are allowed to vary in the range of 408~414 nm and 420~445 nm due to the spatial variation of the PL spectra. The reason to fix the wavelength of the two major PL peaks is that they correspond to the dominant PL emission wavelengths of the two In_x_Ga_1−x_N quantum well groups with designed indium composition (i.e. x = 0.08 and 0.15). As can be seen in Fig. 4(a,b), these two major PL peaks provide the main contribution to the PL spectrum in the green area, which covers a large fraction of the image. However, in the red and black areas, two additional minor PL peaks with blue-shifted wavelengths from those of the major peaks are required to fit the obtained PL spectra well. The necessity of adding these two blue-shifted minor PL peaks to the PL curve-fitting form indicates that the spatial variation of indium composition can now be investigated in detail.

Figure 4(a) is a RGB image that shows the PL peak intensity mapping with spectral information represented by two different colors, where the green intensity corresponds to the intensity of the major PL peak fixed at 450 nm (green curve in Fig. 4(b)), and the red intensity represents the intensity of the minor PL peak between 420 and 445 nm (red curve in Fig. 4(b)). We focus our discussion below on this pair of major and minor peaks that corresponds to the main 450 nm feature of the overall spectra, since the other pair of major and minor peaks show similar behavior (see Supplementary Fig. S1). The typical PL emission spectrum for the green area is shown in the top panel of Fig. 4(b), where the contribution of the main PL feature comes from the 450 nm major peak. In contrast, the typical PL emission spectrum for the red area shown in the middle panel of Fig. 4(b), has a more significant contribution from the minor peak (red curve). Note that the simultaneous presence of both major and minor PL peaks indicates that the spatial fluctuation of indium composition still occurs at a length scale below the spatial resolution of our optical microscope. However, in sub-micrometer scale, our PL spectral mapping clearly shows that the integrated PL intensity in the green area is much higher than that of the red area. The minor PL peak becomes even more blue-shifted and has a much lower PL intensity in the black area.

The PL peak intensity pattern shown in Fig. 4(a) strongly suggests the spatial variation of indium composition in the designed In_x_Ga_1−x_N quantum well groups can be directly related to the PSS-induced residual strain distribution in the MQW layer as shown in Fig. 3(b). For examples, the pattern shown by the green area is similar to the total PL intensity mapping, which was shown in Fig. 2 to be located mainly above the cone area of PSS, while the red and black areas are located above the flat areas around the cones of PSS. The smaller strain for the green area (area above cone), and the larger strain for the red and black area (area above the flat areas of PSS) can also be seen in the variation of the E_2_(high) peak position shown in Fig. 3(d). The small dark points in the green regions are identified as V-shaped pits, where non-radiative recombination dominates. In summary, the InGaN/GaN MQWs above the cone area exhibit longer emission wavelength, brighter PL emission intensity and smaller residual strain. The longer emission wavelength likely results from a higher indium composition in the MQWs above the cone region, and the higher indium incorporation can be attributed to the lattice-pulling effect in the smaller compressive strain growth condition. Consequently, the MQWs above the cone area have a smaller residual strain in comparison to that of the flat PSS area, and benefit from a less severe QCSE and a higher radiative recombination rate. Although the strain variations are derived from the Raman measurements of the GaN epilayer, the thin InGaN quantum well layer has a similar strain variation as the surrounding GaN barrier layer^31^. In the greater compressive strain area above the flat area of PSS, stronger QCSE is expected to cause a red-shift of the PL spectra^32^, contrary to our observation. However, the effect of QCSE on PL emission (ΔE < 10 meV) is much smaller than the effect of indium component fluctuation (ΔE~100 meV), so the strain-induced indium component fluctuation dominates PL spectrum, and leads to more blue-shifted spectra above the flat area of the PSS. Meanwhile, the smaller effective bandgap at the cone area would lead to local potential minima. Therefore, carrier localization effect will be enhanced and it will prevent carrier diffusion to the defects and dislocations. These two effects simultaneously explain the higher PL emission intensity of the MQWs above the cone area. Although Figs 1(c) and 3(b) show that V-pits and dislocations are also located mostly above the cone area, the formation of V-pits can give rise to barriers in the sidewall of V-pits and self-screen the carrier leakage process^23^. The exposure site of edge type dislocation in GaN has also been reported to enhance indium incorporation^33^. However, since the area where the major PL peak is large (green area in Fig. 4(a)) covers a wider region than just around the V-pits, we believe the indium incorporation enhancement is not localized around the V-pits and is mainly strain-induced. Detailed study of the carrier dynamics can also be demonstrated through theoretical simulation. Figure 4(c) shows the strain-related effective indium composition map of the MQWs, which is constructed based on the PL peak wavelength and intensity mapping of the major and minor PL peak for the 450 nm feature. We first determined the concentration of indium corresponding to each of these two peaks, then calculated their averaged concentration weighted by their peak intensity^34^. To model the carrier diffusion, localization and recombination, we applied 2D FEM based Poisson and drift-diffusion solver to calculate the carrier transport and recombination behavior. We setup the material parameters according to the indium map and assume that the electron and holes are generated by the absorption of optical excitation. Finally, the Poisson and drift-diffusion equations were solved self-consistently to obtain the in-plane carrier diffusion and radiative recombination distribution^35^,^36^. As shown in Fig. 4(d), the carriers in general accumulate at areas with higher indium composition, resulting in an enhanced recombination rate. However, due to the different potential landscape distribution that can limit the amount of carriers diffusing into different valleys, different locations with the same indium composition do not typically have the same radiative recombination rate. Specifically, the upper half of Fig. 4(c) shows more areas containing low indium composition, and the carriers increasingly diffuse into the high indium composition areas in the upper half of Fig. 4(d). As a result these high indium areas show particularly large radiative recombination rates. In the same areas, larger intensity was also observed in the PL intensity map (see Fig. S2 in the Supplementary Materials). In contrast, the indium composition map (Fig. 4(c)) used for the starting parameter of the simulation shows only similarly high indium composition for both the upper and lower areas of the image. Therefore, the carrier diffusion and recombination simulation model reveals how variations in the indium composition resulted from the PSS-induced residual strain distribution can lead to the spatial distribution of the observed PL intensity. Visualization of the correlation between the experimental PL intensity mapping and the simulated radiative recombination rate mapping can be found in Fig. S2 of the Supplementary Materials.

## Conclusion

In this paper, we demonstrated that 3D spatial-resolved confocal Raman and PL spectromicroscopy is a promising technique for nondestructive investigation of the optical properties of GaN-based LED grown on PSS. The 3D strain distribution and PL emission property of GaN-based LED were measured systematically. We concluded that the obtained Raman mapping and PL mapping are both correlated to the 3D strain distribution originated from the PSS-GaN heterointerface. In particular, the residual strain in the InGaN/GaN MQWs active layer significantly influences the indium composition distribution and leads to variations in the PL peak position and intensity. The simulated radiative recombination rate was also carried out and showed good agreement with the experimental PL mapping results. Based on the above conclusion remarks, we believe that a sophisticated design of PSS and optimized GaN epilayer growth procedure can significantly reduce the droop effect and improve the performance of GaN-based LEDs.

## Additional Information

**How to cite this article:** Li, H. *et al*. Three dimensional characterization of GaN-based light emitting diode grown on patterned sapphire substrate by confocal Raman and photoluminescence spectromicroscopy. *Sci. Rep.* **7**, 45519; doi: 10.1038/srep45519 (2017).

**Publisher's note:** Springer Nature remains neutral with regard to jurisdictional claims in published maps and institutional affiliations.

## Supplementary Material

Supplementary Information
