# Supplementary material for: Three dimensional characterization of GaN-based light emitting diode grown on patterned sapphire substrate by confocal Raman and photoluminescence spectromicroscopy
Source: Sci Rep. 2017 Mar 30;7:45519. doi: 10.1038/srep45519 (PMC5371985; doi:10.1038/srep45519)
Supplement: Supplementary Information [file srep45519-s1.doc]

**Three dimensional characterization of GaN-based light emitting diode grown on patterned sapphire substrate by confocal Raman and photoluminescence spectromicroscopy**

# Heng Li^1^,, Hui-Yu Cheng^2^, Wei-Liang Chen^2^, Yi-Hsin Huang^2^, Chi-Kang Li^3^, Chiao-Yun Chang^1^, Yuh-Renn Wu^3^, Tien-Chang Lu^1^, and Yu-Ming Chang^2^

^1^ Dept. of Photonics & Institute of Electro-Optical Engineering, National Chiao Tung University, Hsinchu 30050, Taiwan;

^2^ Center for Condensed Matter Sciences, National Taiwan University, 10617, Taipei, Taiwan

^3^Graduate Institute of Photonics and Optoelectronics and Department of Electrical Engineering, National Taiwan University, Taipei, 10617, Taiwan

Supplementary Materials

1. More detail information about the PL peak fitting results shown in Figure 4 (a)
2. Visualization of the correlation between simulation and PL measurement

**More detail information about the PL peak fitting results shown in Figure 4 (a)**

Figure 4(a) of the main text shows the combined intensity mapping of the major-minor peak pair for the 450nm PL feature. To more clearly show the individual peak's contribution, we show the major and minor peak mapping separately for the 450 nm PL feature in the supplementary Fig. S1(a) and S1(b), together with the combined image in Fig. S1(c). The calibration bar for the two peak's mapping are also displayed to show their corresponding range. Figure S1(d), S1(e), and S1(f) are similar images for the major-minor peak pair of the 415 nm PL feature. Comparison of Fig. S1(c) and Fig. S1(f) show that the both pairs of major-minor peak intensities have similar spatial distribution.


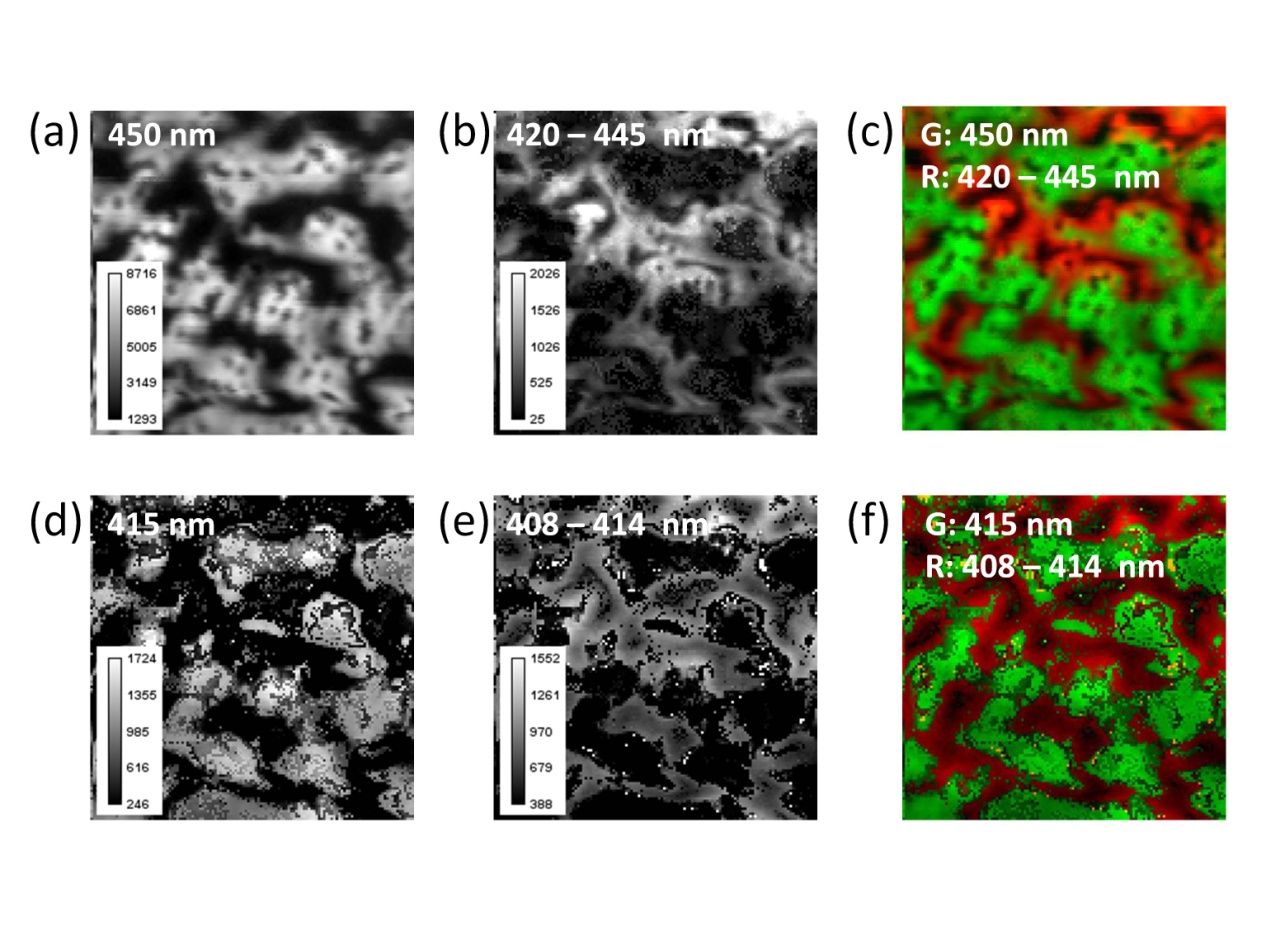


**Figure S1.** Intensity mapping of (a) major and (b) minor peak at 450 nm and 420 - 445 nm. (c) The combined RGB image where red and green are the intensity shown in (a) and (b) respectively. (d) - (f) Similar images for the 415 nm major and 408 - 414 nm minor peak.

**Visualization of the correlation between simulation and PL measurement**

In order to more clearly visualize the correlation between the simulation result with the experimental PL measurement, we show the integrated PL intensity map for the sum of 450 nm major and minor peak profiles in Fig. S2(a). Meanwhile, the simulated radiative recombination rate map in Fig. S2(b) is the same result as Fig. 4(d), but shown on a linear scale to facilitate the visualization of the correlation.


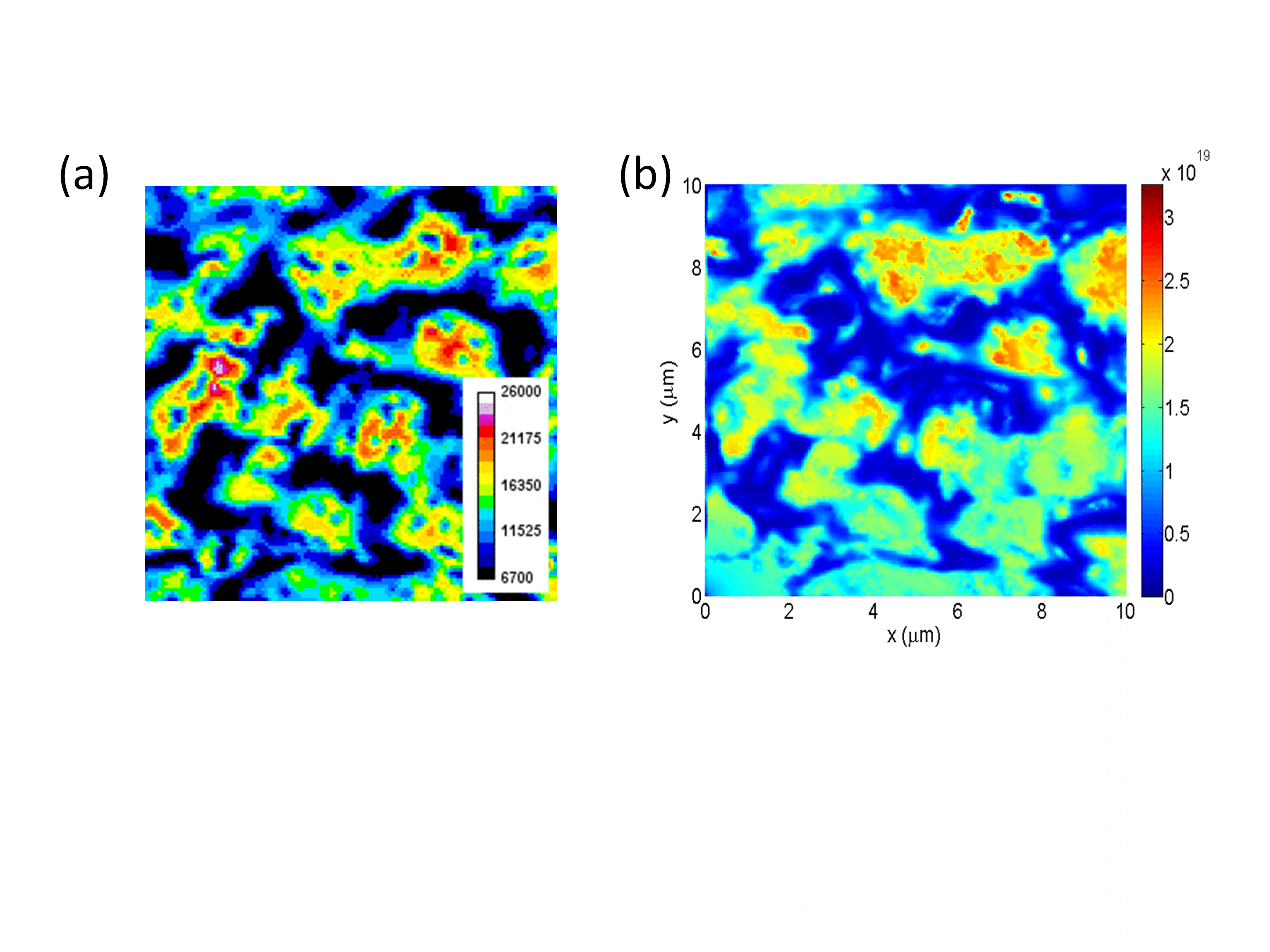


**Figure S2.** (a) Integrated PL intensity mapping for the sum of major peak at 450 nm and its associated minor peak. (b) Simulated radiative recombination rate mapping in the MQWs shown on a linear scale. The unit for the values shown on the scale bar is cm^-3^s^-1^.
